# Age-Related Decline in Controlled Retrieval: The Role of the PFC and Sleep

**DOI:** 10.1155/2012/624795

**Published:** 2012-08-28

**Authors:** Kristine A. Wilckens, Kirk I. Erickson, Mark E. Wheeler

**Affiliations:** ^1^Department of Psychology, University of Pittsburgh, Pittsburgh, PA 15260, USA; ^2^Learning Research and Development Center, University of Pittsburgh, Pittsburgh, PA 15260, USA; ^3^The Center for the Neural Basis of Cognition, University of Pittsburgh, Pittsburgh, PA 15260, USA

## Abstract

Age-related cognitive impairments often include difficulty retrieving memories, particularly those that rely on executive control. In this paper we discuss the influence of the prefrontal cortex on memory retrieval, and the specific memory processes associated with the prefrontal cortex that decline in late adulthood. We conclude that preretrieval processes associated with preparation to make a memory judgment are impaired, leading to greater reliance on postretrieval processes. This is consistent with the view that impairments in executive control significantly contribute to deficits in controlled retrieval. Finally, we discuss age-related changes in sleep as a potential mechanism that contributes to deficiencies in executive control that are important for efficient retrieval. The sleep literature points to the importance of slow-wave sleep in restoration of prefrontal cortex function. Given that slow-wave sleep significantly declines with age, we hypothesize that age-related changes in slow-wave sleep could mediate age-related decline in executive control, manifesting a robust deficit in controlled memory retrieval processes. Interventions, like physical activity, that improve sleep could be effective methods to enhance controlled memory processes in late life.

## 1. Introduction

Age-related cognitive impairments often include decline in executive control critical for strategic controlled memory retrieval [1–4]. Volumetric studies have suggested that atrophy of the prefrontal cortex (PFC) mediates age-related decline in executive control [5, 6]. Impairments in executive control include difficulties selecting relevant and inhibiting irrelevant information and actions, and difficulties monitoring and updating information [7, 8]. Further, white matter in older adults is particularly compromised in anterior brain regions. This has been shown with white matter lesions [9] as well as white matter integrity assessed using diffusion tensor imaging (DTI) [10–12]. Such white matter breakdown disrupts the connectivity between frontal and other cortical regions, ultimately affecting executive control. These PFC changes tend to manifest themselves in a range of cognitive tasks including memory retrieval [3, 13, 14]. Given that decline in memory can be particularly debilitating in older adults, determining factors that contribute to PFC decline is of utmost importance. The prevalence of sleep disruption in older adults [15–17] and its negative impact on cognition [18, 19] suggest that sleep may play an important role in the extent to which older adults exhibit decline in PFC function and in turn, memory retrieval. One compelling implication of this model is that ameliorating sleep deficits in healthy older adults could lead to improvements in PFC function and in turn, memory. 

## 2. Executive Control and Controlled Retrieval: the Role of the PFC

Given the vast amount of information stored in memory, successful goal-directed memory retrieval depends on executive control to select appropriate memories among competing representations [14, 20, 21]. Memory impairments have been shown with task interference during retrieval [22–24], suggesting that some degree of control is required. The critical role of the PFC in executive control during memory retrieval has been demonstrated both with animal models of retrieval [25–27] and neuropsychological studies in patients with frontal lobe damage [28–32]. Complementary evidence comes from neuroimaging studies in healthy young adults, which have consistently shown the involvement of the PFC during memory retrieval [21, 22, 28, 33–35]. Specifically, the PFC is thought to exert executive control on inferior temporal cortex to retrieve stored memories [25]. PFC-mediated executive control is particularly important for memory tasks that require retrieval of details and inhibition of competing or interfering memories [4, 22, 28, 34–36]. These tasks include cued recall, free recall, temporal order memory, and remote memory retrieval [37]. In contrast, the PFC is less important for automatic forms of memory, such as those measured with simple item recognition tasks [28, 37, 38]. Thus, there are distinct retrieval processes that vary in the level of executive control required and dependence on the PFC. 

## 3. Controlled Retrieval and Aging

**Figure 1:**
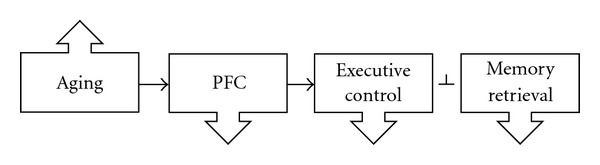
Increasing age leads to decreased prefrontal function, which decreases executive control, in turn blocking (perpendicular line) controlled memory retrieval.

Dissociations in performance on different memory tasks provide evidence that executive control deficits may partly underlie memory deficits in older adults. Older adults show intact recognition memory, a relatively automatic retrieval process, but detailed memory retrieval dependent on the PFC is impaired [4, 33, 39–43]. This is perhaps not surprising because frontal lobe integrity is often affected in aging [6, 44]. This age-related dissociation between recognition memory and detailed memory recall may be driven by insufficient PFC-mediated executive control engaged to constrain the memory search and target relevant details. This sequential relationship between aging, PFC function and executive control, leading to a deficit in controlled retrieval is illustrated in Figure 1.

**Figure 2:**
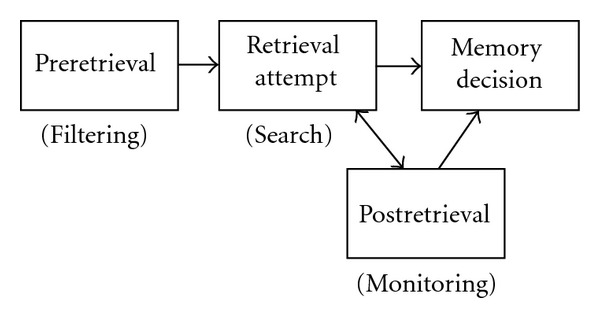
Stages of memory retrieval leading up to a memory decision. Preretrieval processes filter out irrelevant information to constrain the memory search. A retrieval attempt is made. If preretrieval processing is insufficient, postretrieval processing is engaged to edit or elaborate on retrieved content prior to making a memory decision. Bidirectional arrows between retrieval attempt and postretrieval reflect the possibility of additional retrieval attempts prior to the memory decision.

**Figure 3:**
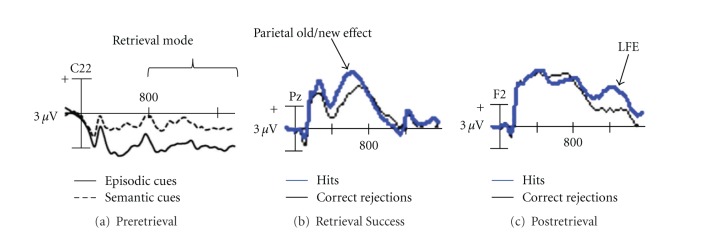
Waveforms reflecting ERP effects associated with three stages of memory retrieval. (a) Preretrieval processing: example “retrieval mode” effect in young adults, from Wilckens et al. [22]. Retrieval mode effects are most robust in anterior and right-sided electrode sites (C22 from a 128-electrode layout shown here). Retrieval mode effects are typically associated with a sustained divergence between ERPs elicited by cues to prepare for episodic memory judgments and semantic memory judgments, starting around 800 ms following task cue onset [22, 23]. (b) Retrieval success: example parietal old/new effect in young adults, from Wolk et al. [49]. The parietal old/new effect is most robust in left posterior electrode sites (Pz from a 35-electrode layout shown here). The parietal old/new effect is associated with more positive-going ERPs elicited by correctly identified studied information (hits) compared with correct rejections (CRs) between 500–800 ms after memory probe onset. The parietal old/new effect reflects successful recovery of a memory trace, often for memory details [58]. (c) Postretrieval processing: example late frontal effect (LFE) in older adults from Wolk et al. [49]. The LFE is most robust in right anterior electrode sites (F2 from a 35-electrode layout shown here). The LFE is associated with more positive-going hits than CRs starting around 1000 ms after memory probe onset and later in right anterior sites [45, 61]. A late-correction strategy would predict that older adults would show diminished retrieval mode (a) and parietal old/new (b) effects, but the LFE (c) would be intact or greater in older adults. Though these effects may be exhibited during simple recognition memory due to spontaneous engagement of retrieval strategies and retrieval of details, these ERP effects are associated more with cue-induced retrieval, rather than stimulus-driven retrieval (i.e., a retrieval mode is engaged across a block of recognition memory judgments suggesting that processing is biased in favor of memory retrieval).

Jacoby and colleagues have posited that while young adults are more likely to rely on an “early selection” memory retrieval strategy, older adults rely more on a “late correction” retrieval strategy [2, 43]. An early selection retrieval strategy engages executive control to filter mnemonic information to be retrieved through selection of relevant information and inhibition of irrelevant information [38]. This is sometimes referred to as *preretrieval processing*. An early selection strategy is often associated with engagement of a retrieval mode (Figure 3(a)), which is a tonic state maintained to ensure stimuli are treated as episodic memory cues [45–48], or as retrieval orientation, which is also a tonic state within retrieval mode, in which stimuli are processed differently depending on the content to be retrieved [23, 45]. In contrast, a late correction strategy may involve *postretrieval processing*, which takes place at the tail end of retrieval to compensate for less efficient preretrieval processing and involves the editing or elaborating on retrieved content for task goals [2]. This may also involve additional retrieval attempts if retrieved content is insufficient to make a memory decision [49, 50]. Postretrieval processing often depends on executive control, albeit less efficient control, later in the retrieval phase [51].  Figure 2 illustrates the series of processing stages leading up to a memory decision reflecting both an early selection and late correction path.

Neuroimaging studies have shed light on how deficits in executive control affect memory in older adults. In addition to recruiting PFC regions typically active during memory retrieval in young adults, older adults also tend to recruit PFC regions not typically active in younger subjects. This pattern of activity is known as “nonselective” recruitment [14]. While young adults tend to show a strong right-lateralized recruitment of the PFC during memory retrieval, older adults tend to also recruit left PFC, and often display an overall decrease in PFC activity [52–54]. One hypothesis is that this nonselective recruitment represents the breakdown of appropriate executive control across cortical regions [14]. This interpretation was supported by Colcombe et al. [55] who showed that nonselective PFC recruitment in older adults was associated with poor inhibitory task performance. Inhibitory control required for preretrieval processing during controlled memory retrieval may be altered with aging and lead to memory deficits.

To investigate whether controlled retrieval in older adults involves insufficient preretrieval processing, Velanova et al. [2] examined age-related differences in controlled retrieval using functional magnetic resonance imaging (fMRI). Velanova and colleagues found that for memory tasks that rely heavily on executive control, older adults showed “nonselective” increased recruitment of frontal control regions (consistent with prior reports [52–54]). This study also investigated temporal properties of the hemodynamic response during retrieval to determine the stage of retrieval in which frontal recruitment took place. The onset of frontal activation in older adults, as measured by a lag in the hemodynamic response, was relatively late in the retrieval phase, following retrieval success effects typically found in parietal cortex [56]. These age-related changes in the onset of the hemodynamic response in frontal regions support the view that retrieval involves different stages of processing contributing to a memory decision. In addition, the observation that frontal activation onset occurred after retrieval success effects in the older group suggests that older adults may rely more on postretrieval processing compared with young adults. 

Using fMRI, Dew et al. [57] investigated whether detailed retrieval-related activity in the medial temporal lobes (MTL) also exhibited an early to late shift in retrieval processing in older adults. They found a reduction in MTL activity in preparation to make a detailed memory judgment, but increased activity late within the retrieval phase in older adults. In addition, they found reduced functional connectivity between the PFC and MTL during retrieval preparation in older adults relative to young, suggesting a deficit in PFC-MTL interactions in anticipation of detailed memory retrieval. 

An event-related potentials (ERP) study conducted by Wolk et al. [49] also found evidence to support this model of cognitive aging. Testing recognition memory, Wolk et al. [49] examined early old/new effects (midfrontal and parietal) typically associated with retrieval success (Figure 3(b)) (see Rugg and Curran [58] for a review of early effects), and late old/new effects (late frontal effect) associated with postretrieval processing in young and older adults (Figure 3(c)). In contrast to prior studies that tested detailed memory retrieval [59, 60], a recognition memory paradigm was used as an “unbiased” form of retrieval such that there were no constraints to successful detailed memory judgments. Retrieval processing reflected both successful and unsuccessful retrieval of details. Compared with young adults, older adults showed diminished early retrieval success effects. However, the late frontal effect, typically associated with postretrieval processing [49, 61–65], was increased in the older group relative to the young. The authors noted that this increased late frontal effect may have served additional retrieval attempts following initial recovery of a weak memory trace given that it occurred after failure to engage early retrieval success effects [49, 50, 66]. An examination of the late frontal effect within the older group demonstrated that this effect was most robust among poor performers, reflecting that this pattern of strategies is primarily evident in older adults with marked memory deficits. 

It should be noted that this study tested recognition memory, which, as a more automatic memory process, is typically spared in older adults and depends less on PFC function and executive control. Postretrieval processing has also been shown to be engaged in both item and detailed recognition memory tasks [51, 67, 68]. In addition, some ERP studies have shown a diminished late frontal effect in older adults relative to young [59, 60], especially when the task requires retrieval of details. One possible explanation for these discrepancies is that older adults overrecruit PFC resources under lower levels of task demands [69], such as in a recognition memory task. In contrast, young adults may more appropriately allocate executive control during cognitively demanding retrieval tasks [69]. Additionally, as mentioned above, postretrieval processing may reflect different processes [45, 49, 50, 66]. Under certain retrieval conditions, early filtering of irrelevant mnemonic information may be insufficient, but the appropriate information is still recovered, so postretrieval processing would involve selecting the relevant information from the retrieved content prior to the memory decision. Alternatively, when early filtering is insufficient, such that the relevant information is not recovered, additional retrieval attempts would be necessary and reflected in later processing. Evidence for the latter comes from studies that show an intact late frontal effect in the absence of accurate memory judgments [50]. Thus, the extent to which postretrieval processing is necessary in young adults may depend on the demands of the task [49]. Reduction in detailed memory retrieval in older adults when the task was limited to detailed memory judgments may contribute to these discrepancies [49]. Regardless of the processes underlying postretrieval processing, the above findings are strong support for the view that frontal overrecruitment displayed by older adults [52–54] may be due to less efficient executive control. Specifically, poor initial engagement of control mechanisms responsible for filtering preretrieval may necessitate additional later control [49], making the retrieval process less efficient. 

The “late selection” model of aging is consistent with other theories of cognitive decline posited outside the realm of memory retrieval [1, 70, 71]. In a review of age-related differences in neural activity associated with top-down modulation of attention, Gazzaley and D'Esposito [71] proposed that normal memory decline is associated with a selective impairment in older adults' ability to inhibit or suppress irrelevant processing, a view originally proposed by Hasher and Zacks [1]. Testing selection and inhibition, they further suggested that older adults' ability to enhance processing related to relevant information is left intact. Gazzaley and colleagues found evidence to support this claim using a variety of neuroimaging techniques. In an fMRI study, Gazzaley et al. [72] investigated enhancement and suppression of neural activity selective for information that was cued as relevant or irrelevant, respectively. Using a working memory task in which young adults were instructed to either remember or ignore faces versus scenes, or passively view either stimulus, Gazzaley and colleagues found that instructions to remember scenes were associated with enhancement of neural activity related to scene processing above baseline in a scene-selective region (left parahippocampal/lingual gyrus), whereas instructions to ignore scenes were associated with reduced scene-selective activity below baseline. In contrast, an older group displayed enhancement of scene-selective neural activity with instructions to remember scenes, but were less likely to show significant suppression activity below baseline with instructions to ignore scenes. This finding suggests that inhibitory processes were impaired in older adults. Relating these findings to behavior, they found that suppression deficits were exhibited only by older adults who were impaired at remembering target stimuli following a working memory delay, and those who were more likely to later remember stimuli they were instructed to ignore. These results revealed that older adults with poor performance were less likely to inhibit task-irrelevant processing. Additionally, although inhibition is associated with right PFC activity in young adults [73], inhibition has also been shown to recruit bilateral PFC in older adults [55, 74] similar to the pattern of results found during memory retrieval. These studies demonstrate robust age-related deficits in inhibitory control. These findings are noteworthy given that inhibition is a critical component of preretrieval processing. 

Though evidence for an age-related decrease in “early selection” has been shown in other areas of cognition [70, 71], there are very few studies that have tested this with episodic memory retrieval. Among the few retrieval studies that have examined preretrieval processing in older adults, they have suggested that older adults are less likely to spontaneously engage in this process [57, 75–78]. For instance, Morcom and Rugg [75] showed using ERPs that while young subjects processed unstudied (new) stimuli differently depending on the type of content to be retrieved (retrieval orientation), this type of processing was diminished among older adults. Accordingly, young adults may have been more likely to constrain their memory search for task-relevant content prior to retrieval.

## 4. Age-Related Changes in Sleep Contribute to Cognitive Decline

Having established that age-related changes in executive control contribute to memory deficits, we can ask which factors result in this specific pattern of impairments. Several factors including sleep have been shown to influence PFC function and executive control in both young and older adults [7, 79–82]. Although sleep behaviors change dramatically with aging (Table 1), sleep has been relatively ignored in studies of normal cognitive decline, especially in the context of controlled retrieval. Research examining the relationship between cognition and age-related changes in sleep is important, however, given the negative impact of sleep deprivation and disruption on cognition [18, 19]. 

In addition to being more sleep-deprived than younger adults, older adults also show a decline in sleep efficiency, sleep continuity, and slow-wave sleep, [15–17, 83–88]. Slow-wave sleep refers to stages 3 and 4 of non-REM (rapid eye movement) sleep measured with polysomnography, an electrophysiological technique to characterize sleep. Slow-wave sleep is characterized by high amplitude, low-frequency delta waves measured with EEG (electroencephalogram) [89]. Importantly, slow-wave sleep is thought to restore PFC function [80, 89], given that from wake to slow-wave sleep, there is significant deactivation in the PFC. Results from PET (positron emission tomography), fMRI, and EEG support this theory [89–93]. Muzur et al. [89] hypothesized that a PFC respite is critical to restore frontal lobe function for wakefulness, which in turn may benefit executive control. Another possibility suggested more recently by Dang-Vu et al. [94] is that slow-wave sleep actively supports frontal lobe function, based on increased activity found during slow-wave sleep in frontal regions relative to baseline non-REM activity. In contrast to prior studies, Dang-Vu et al. [94] compared discrete slow-wave sleep waves with baseline non-REM activity as opposed to wake EEG activity. This lead to the conclusion that slow-wave sleep is not a quiescent state, but rather actively restores brain function. The decrease in slow-wave sleep that is found in late adulthood may contribute to a decline in PFC restoration. This may in turn affect waking PFC function and performance on executive control tasks in older adults.

In the next sections, we will review studies involving sleep and sleep deprivation and how they relate to deficits in controlled retrieval and executive control processes involved in memory retrieval (i.e., inhibition). We propose that age-related decreases in slow-wave sleep may drive age-related changes in PFC function and in turn controlled retrieval.

## 5. Impact of Sleep Deprivation on Executive Control and the PFC

Sleep deprivation influences performance on a variety of cognitive tasks [7, 18, 19]. This outcome does not appear, however, to be explained simply by fatigue or boredom, but by direct effects of sleep deprivation on frontal lobe function, and in turn, cognitive processes that depend on the frontal lobes [7, 80, 89, 95–97]. It is principally the loss of slow-wave sleep that occurs with sleep deprivation that is thought to affect the frontal lobes and underlie the impact of sleep deprivation on executive control [89, 97].

Significant decreases in frontal lobe metabolism have been demonstrated in studies of sleep deprivation [89, 98, 99]. This may be driven particularly by the lack of slow-wave sleep [98]. Moreover, this decrease in metabolism is not fully restored with a full night of recovery sleep [98], suggesting that while a recovery sleep may increase alertness [100], underlying effects on the frontal lobes may persist. 

It is hypothesized that sleep-deprived young adults may serve as an experimental model for age-related cognitive decline [80]. Similarities in patterns of cognitive performance and brain activity between older adults and sleep-deprived young adults support this view [101, 102]. Similar to the pattern of “nonselective” recruitment of PFC found in older adults [2, 52, 53, 55], young adults overrecruit PFC during cognitive tasks following sleep deprivation [101, 102] but see [103–105]. In one of these studies, Drummond et al. [102] had subjects take part in a verbal learning task. Subjects that were sleep-deprived for 36 hours showed increased activation of several “control” regions including the dorsolateral PFC relative to control subjects. As noted by the authors, this pattern of activation was similar to the pattern found in older adults. Similarly, Chee and Choo [101] found with a working memory paradigm that young subjects sleep-deprived for 24 hours showed a pattern of activation and deactivation in parts of frontal and parietal cortex that closely resembled the pattern typically observed in healthy older subjects. They found that while anterior medial frontal and posterior cingulate cortex showed significant deactivation, the left dorsolateral PFC showed an increase with sleep deprivation. This increase in left PFC activity in sleep-deprived subjects certainly parallels the increases found in left PFC in older adults during memory retrieval [53, 54]. These similarities in PFC overrecruitment among healthy older adults and sleep-deprived young adults suggest a common mechanism between sleep disruption and cognitive decline [80]. This common mechanism may be decline in executive control, or broadly PFC function.

Cognitive impairments that arise from sleep deprivation are often found for executive control tasks [7, 102–104, 106–109]. Neuroimaging studies have also provided evidence to suggest that sleep deprivation affects executive control dependent on the frontal lobes [18, 19, 99, 101–104, 107, 110–112]. In contrast to some studies that have failed to demonstrate significant effects of sleep deprivation on cognition using nonexecutive tasks [113], studies using “executive tasks” have demonstrated a specific influence of sleep on PFC function. Some of these studies have investigated executive processes that are important for controlled memory retrieval, such as inhibition. For example, Breimhorst et al. [107] and Schapkin et al. [110] used a Go-NoGo paradigm to test the hypothesis that inhibitory processing is impaired with sleep disruption. This task requires inhibition on NoGo trials when subjects are instructed to inhibit their response to distracters. Using noise to disrupt sleep in young individuals, Schapkin et al. [110] examined ERPs associated with Go and NoGo trials. They showed that the fronto-central P3 amplitude (a positive wave with a 300 ms peak latency) elicited by NoGo trials was reduced in the sleep disruption condition. However, the P3 elicited by Go trials was not affected by sleep disruption. The authors concluded based on these results that the decision process associated with Go trials was not influenced by sleep disruption. However, inhibitory processing associated with NoGo trials was negatively affected. This finding suggests that inhibitory control is impaired with sleep disruption.

Also testing a Go-NoGo paradigm in young adults, Breimhorst et al. [107] examined Go-NoGo ERP effects in good and poor sleepers based on an objective sleep disturbance index using polysomnography. Breimhorst et al. [107] also found that the NoGo P3 latency was longer in poor sleepers relative to good sleepers, reflecting deficient inhibitory processing. However, in contrast to the Schapkin et al. [110] study, Breimhorst et al. [107] also found decreased Go P3 amplitude in poor sleepers. This suggests that poor sleep also affected task-relevant processing, not just inhibition. Despite these differences, these studies collectively suggest that inhibitory processes are negatively impacted by sleep disruption. 

Sleep deprivation also appears to influence task-switching processes. Task-switching involves cognitive flexibility and inhibition of irrelevant task-sets and is often considered a model paradigm of executive control [114, 115]. Couyoumdjian et al. [116] found a significant increase in switch-costs with sleep deprivation in young adults. Importantly, this effect was driven by an increase in response times on switch trials. There was no change in response times on repeat trials, suggesting that sleep deprivation does not globally influence response time. Instead, it specifically affects subjects' ability to switch between task-sets. Also using a task-switching paradigm, Heuer et al. [117] found deficits with task-switching following sleep deprivation. In this study, the task-switching costs were influenced by sleep deprivation only when subjects switched between two tasks as opposed to two stimulus-response mappings. This dissociation may support the view that sleep-deprivation influences inhibition of competing task-sets—a more internal implementation of inhibitory control, important for controlled memory retrieval. 

Harrison and Horne [118] revealed marked impairments on a short and entertaining test of inhibition shown to have a PFC-focus (the Haylings test [119]) following 36 hours of sleep deprivation. This study demonstrated that it was not the tedium of the task that brought about sleep-related deficits, but rather the putative impairments in PFC-mediated inhibition. 

Although multiple aspects of sleep deprivation, including a lack of all sleep stages, and increased stress and fatigue on the part of the subject, may contribute to these impairments, the lack of slow-wave sleep affecting PFC restoration is a possible mechanism by which these impairments in executive control occur [98]. Together, these behavioral and neuroimaging investigations suggest that sleep deprivation has a considerable impact on executive control. We can next ask whether executive control impairments caused by sleep deprivation impact memory retrieval. 

The role of sleep in the offline strengthening of memories through consolidation and integration is a well-established phenomenon in both humans and animals [120–123], and this process may also be vulnerable to age-related decline [81]. Sleep-dependent consolidation, however, will not be discussed in depth here because the present paper is focused on strategic memory processing following sleep (during retrieval) as opposed to memory processing during sleep (consolidation), and because a number of thorough reviews already exist in the literature on sleep and consolidation [81, 120, 124–128]. 

In terms of episodic memory, there are very few studies that have investigated how different retrieval strategies and stages are influenced by sleep. Nonetheless, these studies have demonstrated specific impairments in retrieval processes that depend on the PFC (Table 2). Harrison and Horne [129] examined both recognition memory and temporal order memory judgments. Following a period of sleep or sleep deprivation, subjects were asked to identify whether faces were presented at study or not (recognition) and to make a recency judgment by identifying on which of two study lists the face appeared (temporal order). This temporal order task was posited to depend on the PFC. They found that while recognition memory was left intact, temporal order memory was significantly impaired following sleep deprivation. Using a verbal learning task, Drummond et al. [102] found that recall, but not recognition performance decreased with sleep deprivation. FMRI data collected in this study found increased PFC recruitment during encoding following sleep deprivation. However, the retrieval phase was not scanned in this particular paradigm. So it is unclear whether PFC overrecruitment occurred during retrieval as well following sleep deprivation. These findings suggest that sleep affects controlled retrieval tasks like recall, but leaves more automatic retrieval processes intact.

Supporting the notion that memory deficits in older adults and sleep-deprived young adults are similar, Nilsson et al. [130] found similarities in memory performance between older, young alcohol intoxicated, and young sleep-deprived subjects. In a recall test using weakly and strongly related word pairs, they found that all experimental groups (older, intoxicated, and sleep-deprived) demonstrated the same pattern of deficits in which recall of weakly related word pairs was significantly lower than that of the control young adult group. Recall of strongly related word pairs, however was not affected. In this study, recall of weakly related word pairs should require greater reliance on executive control than recall of strongly related word pairs. The authors attributed this finding to both deficient encoding and retrieval and suggested a functional similarity between sleep deprivation, intoxication, and normal aging in terms of controlled memory processes. 

Recognition memory is primarily uninfluenced by sleep deprivation, however similar to frontal lobe patients [28, 30, 32, 131] and older adults [132, 133], false recognition to semantically related lures has been shown to increase with sleep deprivation (Table 2). Diekelmann et al. [134] used a false memory paradigm [135] to test false recognition in young subjects sleep deprived during memory retrieval. Subjects sleep deprived during memory retrieval were more likely to incorrectly judge new words semantically related to studied words as “old.” This suggests that forms of recognition memory that depend on the PFC (distinguishing semantically-related lures from studied items) are influenced by sleep deprivation. To further support the view that this effect was not a result of less consistent memory consolidation, this study found that manipulations in sleep within the study-test interval did not influence false recognition. It was specifically the effect of sleep deprivation on retrieval that brought about an increase in false recognition. 

Mograss et al. [104, 105] investigated how ERP old/new effects were influenced by sleep deprivation. They found that the late frontal effect was diminished following total sleep deprivation. This effect is attributable to insufficient PFC function, leading to insufficient retrieval of details, supporting the view that sleep deprivation interferes with PFC function during controlled memory retrieval. If retrieval impairments involve inappropriate allocation of PFC resources, sleep deprivation may have resulted in the misallocation of PFC resources manifested by an *under-recruitment *of PFC. 

Overall these sleep-deprivation studies of memory retrieval suggest that more controlled retrieval processes are impaired with sleep deprivation compared with more automatic ones and this dissociation may be driven by a breakdown in PFC function.

These results point to the possibility that age-related decreases in sleep contribute to executive control deficits. Conversely, because there is more to age-related sleep changes than a mere overall decrease in sleep, the impact of age-related sleep changes on cognition may not be completely comparable to sleep deprivation in young adults. Further, while many older adults exhibit marked impairments in cognitive performance and changes in brain activity, some older adults show little cognitive decline. These individual differences in cognitive decline may be explained, at least partially, by individual differences in sleep. Determining the way in which sleep plays a role in age-related cognitive decline may shed light on why some older adults but not others exhibit impairments.

## 6. Individual Differences in Sleep and Executive Control in Older Adults

Inadequate sleep is very common among the adult population [136]. According to Mander et al. [137], only 26 percent of adults report getting the recommended eight or more hours of sleep per night. In addition, total sleep deprivation (when subjects are deprived for a full night) is not necessary to reveal significant cognitive impairments. More ecologically valid studies of chronic sleep restriction involving less than 7 hours of sleep per night for multiple nights have revealed a range of cognitive deficits including deficits on tasks of attention and working memory [18, 19, 138]. Moreover, chronic sleep restriction for two weeks has been shown to result in cognitive deficits equivalent to that found with total sleep deprivation [138]. This type of chronic inadequate sleep, which is similar to sleep behavior of older adults, could potentially result in cognitive impairments that may be difficult to reverse with a few good nights of sleep. Although the data are somewhat inconsistent, older adults with greater sleep quantity and quality tend to perform better on cognitive tasks [79, 81, 82]. 

**Figure 4:**
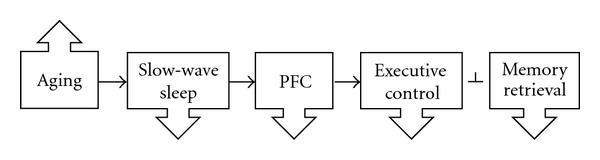
Increasing age leads to decline in slow-wave sleep, and in turn, decreased prefrontal restoration. This decreases executive control abilities, which blocks (perpendicular line) controlled memory retrieval.

Given the importance of slow-wave sleep in PFC restoration, decline in slow-wave sleep is a critical age-related sleep change that may contribute to impairments in executive control relevant for memory retrieval. Both human and animal studies have shown a decrease in slow-wave sleep with age [15, 16, 81, 83, 84, 87, 139–142]. This decline in slow-wave sleep gradually manifests itself during the middle years of life [84, 139, 141]. It is possible that the decrease in slow-wave sleep that occurs with aging could negatively impact PFC function by diminishing the restoration process. In addition, older adults that exhibit reduced slow-wave sleep may be more likely to exhibit cognitive decline. This relationship leading to decline in memory retrieval is illustrated in Figure 4. Alternatively, the proposed pathways illustrated in Figure 4 may be neither unidirectional nor an exhaustive model of moderators and possible mediators involved in age-related deficits. For example, amyloid deposition has been shown to disrupt slow-wave sleep [143]. Accordingly, retrieval abilities may be related to slow-wave sleep as a result of age-related neuropathological changes negatively impacting slow-wave sleep. 

It should be noted that several reports suggest that older adults are more resilient to sleep deprivation than young adults [86, 144], suggesting that sleep need declines with age. However, other studies suggest that young and older adults require the same amount of sleep, and time spent in slow-wave sleep to perform well on executive control tasks [145]. Moreover, older adults may be less likely to restore frontal lobe function following sleep deprivation compared with young adults [146]. Despite these age differences in responses to sleep deprivation, it is unclear from these studies whether sleep normally exhibited by older adults negatively impacts cognitive performance. Although there is a dearth of research on the topic, examining whether individual differences in sleep among older adults explain variation in memory and cognitive function is essential given the preponderance of sleep and cognition-related problems among older adults. 

A few studies have suggested that individual differences in slow-wave sleep are related to executive control abilities in older adults. Anderson and Horne [147] examined low-frequency delta EEG activity during non-REM sleep, which is highest during slow-wave sleep, in a group of healthy older adults. They found a positive correlation between low-frequency delta activity in frontal EEG sites and performance on cognitive tasks thought to be relatively “PFC-specific,” including the Wisconsin Card-Sorting Task, and the Tower of London task (a nonverbal planning task). According to the authors, the nonverbal planning task required flexibility in planning and in changing of strategies. The Wisconsin card sorting task is thought to depend on inhibitory control (and other processing) in that it tests for perseveration of strategies. Though this study was purely behavioral, this result points to the relationship between slow-wave sleep and PFC function among older adults. 

In an earlier study, Crenshaw and Edinger [148] investigated whether slow-wave sleep was related to performance on “simple reaction time” and vigilance tasks among older adults with normal sleep and those with insomnia. Older adults who were normal sleepers showed no relationship between cognitive performance and slow-wave sleep. In contrast to the Anderson and Horne [147] study mentioned above, the cognitive measures of this study were not “executive tasks.” Based on the view that slow-wave sleep specifically affects PFC function responsible for executive control, slow-wave sleep would not be related to behavior on these tasks in healthy older adults. Accordingly, the Anderson and Horne [147] study measured simple response time as well and found no relationship between this cognitive measure and slow-wave sleep.

To corroborate these findings, a more recent study, Nebes et al. [79] showed that subjective poor sleep in older adults was associated with poor performance on a range of executive control tasks, including those that test working memory and attentional set shifting. There was, however, no relationship between sleep quality and a processing speed task, supporting the view that sleep does not influence nonexecutive tasks. Though, in this study there was not a clear distinction between executive and nonexecutive tasks: no relationship was found between sleep and inhibitory processing as assessed by the Stroop task and Haylings task, or episodic memory, as assessed by the logical memory test [149]. Regardless of the lack of a clear distinction, these studies examining individual differences in sleep suggest that poor sleep, particularly slow-wave sleep, in older adults may lead to poor performance on some tasks of executive control. It should also be noted that subjective sleep quality, as measured by the Pittsburgh Sleep Quality Index [150] in the Nebes et al. study, primarily measures sleep quality based on time spent in bed, as opposed to objective amount of time spent sleeping or time spent in specific sleep stages. Consequently, relationships between sleep and these cognitive tasks may have differed if objective sleep measures, such as amount of time spent in slow-wave sleep, were investigated.

Based on the studies reviewed above, there is clearly some support for the hypothesis that age-related changes in sleep contribute to decline in PFC and executive control and this may affect controlled memory abilities. Should future research support this model of cognitive aging, treatments aimed at improving slow-wave sleep in healthy older adults could improve executive control, potentially leading to improvements in memory. 

Sleep disruption and slow-wave sleep in young and older adults appear to have the capacity to influence controlled memory retrieval. However, the influence of these factors on controlled retrieval remains unclear. Future research should examine whether pre and postretrieval processing are differentially influenced by sleep deprivation, sleep treatment, or individual differences in slow-wave sleep in recall and recognition memory paradigms. This would answer the question of whether the mechanisms underlying impairments in memory retrieval are similar between older adults and sleep-deprived young adults. Given the “nonselective” frontal recruitment found in both older and sleep-deprived individuals, insufficient inhibitory and filtering processes prior to retrieval is a possible mechanism driving sleep-related impairments in controlled memory retrieval. To the extent that sleep deprivation can be thought of as an experimental model for cognitive aging, we would expect that sleep-deprived subjects would rely more on postretrieval processing, and less on preretrieval processing [2, 49, 76]. Further, it is conceivable that older adults with the least slow-wave sleep would be most likely to exhibit a “late correction” retrieval strategy. 

## 7. Sleep as a Mediating Variable for Effects of Exercise on Cognition

**Figure 5:**
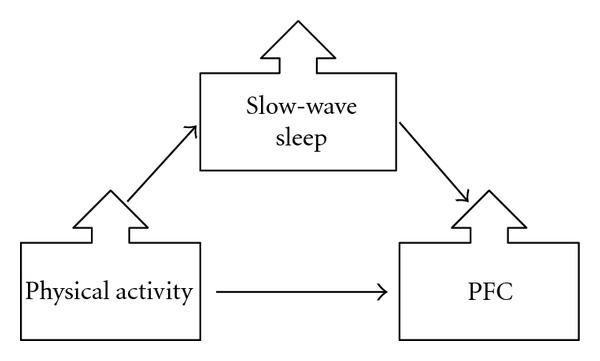
Model illustrating slow-wave sleep as mediating the relationship between physical activity and PFC function. With an increase in physical activity, there is an increase in slow-wave sleep, leading to improved PFC function and in turn improved executive control function.

Having concluded that age-related changes in sleep may contribute to the pattern of cognitive deficits displayed by older adults, we can next ask whether sleep acts as a mediating factor for other variables that influence cognition. For example, physical activity interventions have been shown to improve executive control in both young and older adults [151, 152]. There are consistent benefits of physical activity and exercise interventions on executive control that appear to be mediated by biological markers of brain function [5, 151, 152]. The pathway through which exercise benefits executive control, however, is not well understood [153]. One possibility is that exercise improves cerebral vasculature, thereby influencing cognitive function [154]. Another possible mechanism is that exercise improves sleep, which in turn benefits cognition.  Figure 5 illustrates the possible mediating relationship between sleep, physical activity, and executive control. Reviews of the literature on the relationship between sleep and physical activity suggest that exercise improves both subjective and objective sleep measures, especially in older adults with poor sleep [153, 155, 156]. Older adults who are more physically fit tend to have shorter sleep latencies (time it takes to fall asleep) and more slow-wave sleep than sedentary older adults [153]. Subjective sleep quality has also been shown to improve with chronic exercise [157, 158]. King et al. [158] found that subjective sleep quality in older adults, as measured by the Pittsburgh Sleep Quality Index, improved with 16 weeks of aerobic exercise. Though few studies have used objective sleep measures, particularly polysomnography, to test the effects of chronic exercise on sleep [155], chronic aerobic exercise in sedentary older adults has been shown to selectively improve slow-wave sleep with a 6-month exercise intervention [159]. Although not directly addressing cognition, these findings are noteworthy in that they suggest that exercise might improve PFC function and executive control by improving slow-wave sleep. 

## 8. Clinical Relevance

This paper has focused on the influence of sleep on memory and cognition in healthy aging, but in the interest of informing interventions in healthy older adults, it is worthwhile to acknowledge the efficacy of treating sleep-related disorders and its impact on cognition. Treatment of obstructive sleep apnea syndrome with continuous positive airway pressure has been associated with significant improvements in performance on executive control tasks (see Jones and Harrison [7] for a review). Naegele et al. [160] showed improvements in a range of tasks thought to be sensitive to frontal lobe function, including the Wisconsin card sorting task, the Stroop task, and a long-term visual memory task. Neau et al. [161] found significant improvement in trail making task B [162], which involves task-switching, but not trail making task A. Given that trails B is a more executive task than trails A, treatment to improve disordered sleep may have specific benefits to executive functions and the PFC. 

Patients with Alzheimer's disease often have disturbed sleep, sleep disorders, and exhibit decreased slow-wave sleep [163, 164]. This may be the result of disruption in circadian rhythms [165]. Given that sleep treatments have been effective in improving cognition in nondemented patients with sleep disorders, it is important that future studies investigate whether sleep treatments would aid in improving dementia symptoms in patients with memory disorders. A recent study [166] demonstrated a significant positive relationship between measures of sleep quality and memory performance in patients with mild cognitive impairment—an intermediate stage between normal aging and Alzheimer's disease. This points to the possibility that symptoms of mild cognitive impairment may be alleviated with improvements in sleep. 

## 9. Discussion

Recent behavioral and neuroimaging research has revealed compelling evidence to suggest that memory impairments found in older adults are driven by deficits in executive control. Impairments in controlled retrieval processes, such as those that occur prior to retrieval, may be mediated by general impairments in executive control. This view of cognitive aging is supported by both studies of selection and inhibition [71] in which older adults show impaired suppression of task-irrelevant processing, and long-term episodic memory [2, 57, 76], in which older adults display nonselective recruitment of the PFC late within the retrieval phase. Although future work is needed to determine the mechanisms by which older adults shift to rely on later processing, current studies suggest that older adults recruit PFC resources relatively late in the processing stream under certain retrieval conditions. A combination of ERP and fMRI techniques that maximize temporal and spatial resolution of these processes will aid in examining whether “late correction” strategies involve reliance on later less efficient PFC processing, or simply less PFC processing in general. 

Given that aging is (a) associated with changes in sleep, and (b) that sleep deprivation and aging reveal similar patterns of deficient cognition and brain activity, it is conceivable that sleep-deprived young adults may serve as a model for cognitive deficits found in older adults [80]. Although sleep deprivation consistently reveals significant impairments in executive control, the sleep deprivation literature has also revealed a wide range of cognitive impairments, some consistent and others inconsistent with the pattern of results typically found in older adults. Additionally, some aging studies have used sleep deprivation to examine how poor sleep affects cognition in older adults. This literature points to both increased and decreased cognitive impairments in older adults relative to young [86, 145]. These inconsistencies could be explained by there being different mechanisms underlying cognitive deficits with aging and sleep deprivation. One drawback to using sleep deprivation as a model for cognitive decline is that slow-wave sleep is the sleep stage that most reliably shows age-related changes [81] and appears to restore PFC function [89]. Total sleep deprivation studies, however, deprive subjects of both REM and non-REM sleep. Further, sleep deprivation is known to influence not only the PFC and “control regions”, but other brain regions including the thalamus, and in turn alertness [98]. Thus, sleep deprivation's influence on cognition may not be specific to executive control. 

The way in which age-related decreases in slow-wave sleep affect the PFC may shed light on whether similar mechanisms underlie cognitive deficits resulting from aging and sleep deprivation. For instance, age-related changes in slow-wave sleep may have an immediate or gradual cumulative impact on PFC structure and function. It may be that chronic diminished slow-wave sleep over time brings about changes in the PFC seen with advanced age. Alternatively age-related reductions in slow-wave sleep could have an immediate effect, similar to experimentally induced sleep deprivation. Examination of individual differences in slow-wave sleep among healthy older adults may prove effective in revealing the specific processes influenced by age-related changes in sleep and more directly address whether age-related changes in sleep robustly affect cognitive decline. 

Overall, given that older adults show a wide range of variability in cognitive performance, it is necessary to determine whether there is also a wide range of variability in sleep changes associated with age, and whether the individual differences in cognition and sleep covary. The results from these studies may inform interventions aimed at preventing or reversing cognitive decline. Intervention studies with sleep treatments may address whether age-related changes in sleep directly affect cognition in older adults. Further, exercise intervention studies examining effects on both sleep and cognition may answer questions regarding the mechanisms underlying the relationship between exercise and cognition. These interventions may also serve as a useful technique for improving sleep-related cognitive decline in older adults. 

## Glossary

 
*Old/new effects*. Differences between brain activity elicited by correctly categorized studied items and brain activity elicited by correctly categorized new items presented at test.
 
*Retrieval success*. Refers to old/new effects typically found in parietal regions reflecting successful memory recovery.
 
*Retrieval attempt*. Memory search process prior to the memory decision.
 
*Retrieval orientation*. The differential processing of retrieval cues based on the sought after information to maximize retrieval success.
 
*Slow-wave sleep*. Non-REM sleep stages (3 and 4) in which low-frequency delta EEG activity is the highest.
 
*Sleep quality*. Sleep measures based on time spent lying in bed, including sleep efficiency (proportion of time spent lying down asleep), wake after sleep onset and sleep latency (time it takes to fall asleep).
 
*Sleep efficiency*. Proportion of time spent asleep versus time spent lying down.
 
*Executive control*. Mechanism responsible for goal-oriented processes that involve selection of relevant and inhibition of irrelevant information and actions and the monitoring and updating of information.
 
*Preretrieval processing*. Processing that takes place in preparation for memory retrieval to filter irrelevant mnemonic information and constrain the memory search space.

 
*Postretrieval processing*. The monitoring or updating of information following retrieval as relevant for task demands.
 
*Episodic memory retrieval*. Recovery of memories for personally experienced events usually involving some recollection of details. In an experimental paradigm, studied information must exceed working memory capacity and be cleared from working memory prior to retrieval.
 
*Semantic memory retrieval*. Recovery of information from knowledge about the world (i.e., living/non-living judgment).
 
*Obstructive sleep apnea syndrome*. Sleep disorder characterized by frequent breathing cessation leading to brief arousals.

## Figures and Tables

**Table 1:** Consistent sleep changes reported from young to older adulthood.

Total sleep time (decrease) [15, 17, 81, 167]
Sleep efficiency (decrease) [15, 17, 83]
Wake after sleep onset (WASO) (increase) [15, 17, 167]
Slow-wave sleep (decrease) [15, 16, 83–85, 139–141, 167]

**Table 2:** Retrieval processes influenced by advanced aging, PFC damage, and sleep deprivation. Simple item recognition is thought not to depend on the PFC except under conditions in which subjects are required to distinguish between studied items and unstudied items semantically related to studied items (false recognition). Some effects of aging, PFC, and sleep deprivation on these memory processes are attributable to impairments in both retrieval as well as encoding strategies.

Cued recall
Aging [168, 169]
Frontal lobe damage [32]
Sleep deprivation [130, 170]
False recognition (False alarming to related lures)
Aging [132, 133]
Frontal lobe damage [28, 30, 32, 131]
Sleep deprivation [134]
Free recall
Aging [133, 171]
Frontal lobe damage [28, 172, 173]
Sleep deprivation [102, 174]
Temporal order memory
Aging [67, 175, 176]
Frontal lobe damage [177, 178]
Sleep deprivation [129]
